# Meloxicam and Buprenorphine Treatment after Ovarian Transplantation Does Not Affect Estrous Cyclicity and Follicular Integrity in Aged CBA/J Mice

**DOI:** 10.1371/journal.pone.0106013

**Published:** 2014-08-25

**Authors:** Anna H. Le, Luis A. Bonachea, Shelley L. Cargill

**Affiliations:** Department of Biological Sciences, San José State University, San José, California, United States of America; National Cancer Institute, United States of America

## Abstract

Angiogenesis, the formation of new blood vessels, is important for the survival of ovarian transplants and the restoration of ovarian functions. Without angiogenesis, transplanted ovarian tissue becomes more susceptible to tissue damage and necrosis. Administration of analgesics for pain management has been shown to decrease angiogenesis, which can influence transplant success especially in aged animals. Aging and the effects of hypoxia after transplantation decrease reproductive viability of the ovarian transplant; therefore, it is important to understand the additional effects of analgesics on aged animal models. The present study investigated the effects of two analgesics, buprenorphine, an opiate, and meloxicam, a non-steroidal anti-inflammatory drug (NSAID), on the reproductive indicators related to estrous cyclicity and follicular integrity after ovarian transplantation of young ovaries into aged CBA/J mice. These aged females did not show any different reproductive responses when treated with either buprenorphine or meloxicam. No significant differences were observed in estrous cycle length, the onset of estrous cycling, the regularity of estrous cycles, and the proportion of viable follicles and total number of follicles per ovarian sample across treatment groups.

## Introduction

Angiogenesis, the development of new blood vessels, is important during normal tissue development and healing. Angiogenesis, therefore, is an important determinant in the outcomes of organ transplantation as tissues require a continuous supply of nutrients, oxygen, and hormones as well as a route for removal of wastes in order to maintain viability. Angiogenic processes in normal tissue, as opposed to tumors, decrease into adulthood but occur regularly in the adult female reproductive system [1], [2]. In female rodents, angiogenesis is particularly important for the estrous cycle, which regulates varying levels of estrogen and progesterone for ovarian functions [3], [4], [5].

While the use of analgesics is advised in survival surgeries to minimize pain and discomfort in research animals, analgesics can reduce angiogenesis [6], [7], [8], [9], [10]. Although not all of the interactions of analgesics and angiogenesis have been elucidated, the putative anti-angiogenic effects of the two classes of analgesics, opiates and NSAIDs, have been investigated in some *in vivo* and *in vitro* studies [6], [7], [8], [9], [10].

In a previous aging study, the transplantation of ovaries from young CBA mice into aged, late-reproductive female mice significantly increased the remaining life expectancy of the recipients [11]. In that experiment, nearly all transplantations performed were successful, as was indicated by the restoration of estrous cyclicity. Further experiments were performed, with all procedural details kept consistent except for the additional use of post-surgery buprenorphine [12]. However, unpublished data from the same experiment suggested several unsuccessful transplants, as indicated by the lack of estrous cyclicity after surgery. It is possible that the post-surgical administration of analgesics negatively influenced transplantation success by decreasing angiogenesis and thereby decreasing the blood supply to the transplant [6], [7], [8], [9], [10].

Aging has long been acknowledged in its role in decreased female fertility [13], [14]. Angiogenesis becomes deficient with age [15] and may negatively influence reproductive function. Aged 40–48 week old female ICR mice showed a higher frequency of oocytes with DNA fragmentation, implying increased apoptotic cells compared with young 7–8 week old mice and 20–24 week old mice [13]. Estrous cycles become extended in aged mice, often leading to the cessation of cycling [14]. In addition to the effects of aging, ischemic injury due to transplantation may cause decreased viability of ovarian transplants [16], [17]. It has been demonstrated that ovarian size and the number of follicles were dramatically decreased after orthotopic grafting in mice [16]. Although mice have also demonstrated the restoration of reproductive cycling after transplantation, distinguishable estrous cycles were not always clear [17]. The use of aged models that are subject to treatment with analgesics for ovarian transplantation may have compounding effects on reproductive function. This highlights the importance of evaluating analgesic effects in aged transplant recipients to understand its impact in future transplantation studies in aged animals.

The effects of two analgesics, buprenorphine and meloxicam, on ovarian transplant success in aged females were evaluated and compared using follicular analysis, ovarian size, and estrous cyclicity post-surgery as indicators of transplant viability. A decrease in the viability of the transplant would indicate decreased angiogenesis [4], [5]. As the two different classes of analgesics have different mechanisms of action, the two analgesics may have different effects on angiogenesis and transplant viability [18], [19], [20].

## Materials and Methods

### Ethics Statement

This study was carried out in strict accordance with the recommendations in the Guide for the Care and Use of Laboratory Animals of the National Institutes of Health. All experimental procedures were approved by the Institutional Animal Care and Use Committee at San Jose State University (Protocol #959). Surgery was performed under sodium pentobarbital anesthesia, and all efforts were made to minimize suffering.

### Mice

Adult CBA/J strain female mice (Jackson Laboratory, Sacramento, CA) were housed under controlled conditions of temperature (21±2°C), humidity (minimum 50%) and lighting (12L:12D, lights on at 0700 hours) in accordance to the University Animal Care guidelines with approval by the San José State University Institutional Animal Care and Use Committee. Animals received feed (Purina Mouse Chow 5008: 23.5% protein, 6.5% fat; Purina Mills. St Louis, MO) and water (deionized) *ad libitum*. Through power analysis, we determined that a sample size of nine focal animals per treatment group would be needed to detect a one-tailed mean difference between groups in the range of 1.5–2 standard deviations from the control animals for a single response. A sample size of 10 animals per group was chosen to account for the roughly 5% transplant failure rate and 5% seizure loss rate observed in previous studies (pers. obs.). For every experimental animal, a donor female was used to supply transplant ovaries, giving a total of 60 female mice anticipated for use in this study. As a note for other researchers, while CBA/J mice are known to be prone to seizures [21], we did not anticipate the high percentage of animals that died of seizures before the end of the study (n = 4, roughly 13%). Prior to surgery, three females died due to seizures and were excluded from the study. As a result, 27 recipient and 27 donor females were used in the transplantation procedures. Recipient and donor females were group housed with 5 animals per 26×17×13-cm cage until surgery. After surgery, each recipient female was housed individually in a 26×17×13-cm cage for the duration of the experiment. Male mice were housed in cages adjacent to female mice to promote estrous cycling in females [22].

### Surgical Procedures and Vaginal Cytology

Bilateral ovariectomies of and ovarian transplantations to recipients at 11 months of age were carried out as previously described [23] with the following exception: the ovarian bursa was closed with one suture of 7-0 Ethilon nylon filament (Ethicon, San Angelo, TX) instead of the 6-0 suture [23]. The transplanted ovaries were from 2-month old females of the same strain [23]. Reproductive data in the form of vaginal cytology were collected via vaginal lavage daily at 0730 hours for one month when the recipient females were 5 months old (pre-surgery), for 14 days immediately prior to ovarian transplantation surgery, and daily starting three days after ovarian transplantation surgery for approximately 75 days (73–76 days) until the end of the experiment. All vaginal cytology data were assessed as wet mounts without staining. Donor mice were housed in close proximity to males for 1–3 weeks prior to use and were at an age considered to be reproductively competent [11] [12]. Donor mice estrous cyclicity was confirmed after surgery, as all ovarian transplant recipients cycled within 10 days after receiving the transplant. At recipient female ovariectomy, complete removal of all recipient ovarian tissue was visually confirmed under stereomicroscopy. At sacrifice, the presence of only transplanted ovarian tissue was visually confirmed under stereomicroscopy, and upon removal for analysis, no residual ovarian fragments from the ovariectomized recipient ovary were observed.

### Analgesic Administration

For administration of drug treatments after surgeries, meloxicam, buprenorphine, and 0.9% saline (no analgesics) were randomly assigned to nine females each. Two saline-treated females died during the course of the experiment. One female died during the transplantation process due to anesthesia complications. CBA/J mice are susceptible to seizures, which may have been the cause of an approximately 1 month post-surgery premature death for the second female [21]. Both of these females from the saline control group were excluded from the study, for a new *n* of seven. After the unplanned losses in the saline group, the final sample size of n = 7 for the saline group would have allowed us to detect a difference as small as 1.9 SD from the mean of the saline group. The meloxicam treatment group received IP meloxicam (Boehringer Ingelheim, St. Joseph, MO) doses of 5 mg/kg of body weight, the buprenorphine treatment group received IP buprenorphine (Reckitt Benckiser Healthcare, Hull, England) doses of 0.05 mg/kg of body weight, and the saline-control group received IP 0.9% saline (Vedco, Inc., St. Joseph, MO) with the same volumes given with analgesic treatments. The analgesic doses administered were in accordance of veterinary recommendations and previous studies [12], [24], [25], [26]. These treatments were administered every 12 hours for 48 hours post-surgery, with the first dose of treatment administered at the end of surgery prior to recovery from anesthesia.

### Fixation and Preservation of Ovaries

All female mice were randomly reassigned a new identification number for subsequent follicular analysis and sacrificed by cervical dislocation. Immediately after sacrifice, one ovary from each female was placed into optimal cutting temperature (OCT) medium (Sakura Finetek, Torrance, CA) and frozen over isopentane (Fisher Scientific, Fair Lawn, NJ) and dry ice (Praxair Distribution Inc., San Jose, CA). The fixed and frozen ovaries were transported overnight by FedEx to IHC World (Ellicott City, MD) to be sectioned using a cryostat. Serial consecutive sections of 6 µm thickness were placed onto glass microscope slides (TruBond, Woodstock, MD) and shipped to San Jose, CA. Upon receipt of the shipment, slides were immediately stored in −80°C until staining with hematoxylin and eosin. Nine consecutive sections per ovary were used for follicular analysis. Analyses made from these nine tissue sections are referred to as a single ovarian sample.

### Histological Staining and Assessment

After removal of slides from −80°C storage, slides were warmed to room temperature, fixed in ice cold acetone (Fisher Scientific, Santa Clara, CA) and rinsed twice in phosphate buffered saline (PBS). The primary stain, Mayer’s hematoxylin (Thermo Fisher Lab Vision, Fremont, CA), was applied for five seconds, then rinsed under running tap water for 30 seconds. Slides were counterstained with 0.5% eosin Y in 95% ethanol (Allied Chemical & Dye Corp, New York, NY) for two minutes and rinsed with distilled water by dipping the slide ten times. Slides were dehydrated using 95% ethanol followed by 100% ethanol (Fisher Scientific, Pittsburgh, PA), cleared using xylene (Fisher Scientific, Fair Lawn, NJ) and a coverslip was applied using SecureMount™ mounting medium (Fisher Scientific, Kalamazoo, MI).

Hematoxylin and eosin stained slides were utilized for determination of follicle numbers and the mean cross sectional area from each ovarian sample. Follicular data were collected from images captured on a Leica ICC550HD camera (JH Technologies, Fremont, CA) that had been fitted on a Leica DM500 microscope (JH Technologies, Fremont, CA) at a total magnification of 100X. Cross sectional area per ovarian sample was measured in ImageJ software (National Institutes of Health, Bethesda, MD).

**Figure 1:**
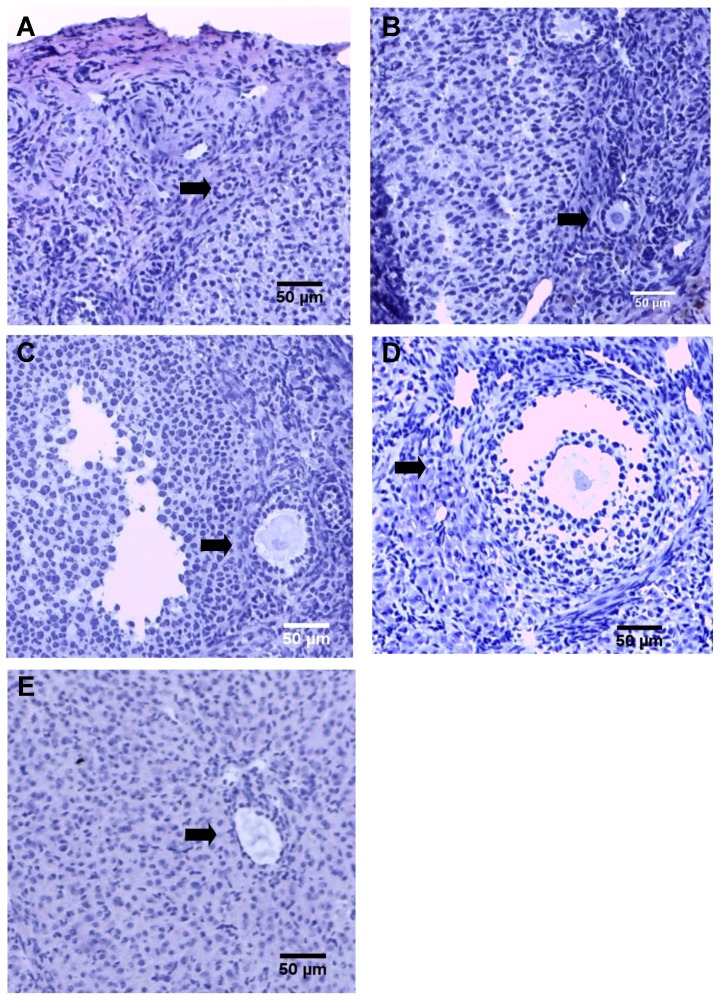
Representative follicle types, H&E stain, 100X total magnification. (A) primordial follicle, (B) primary follicle, (C) secondary follicle, (D) antral follicle, (F) atretic follicle. Corresponding follicles are marked by arrows.

Both viable and atretic follicles were counted from serial sections of each ovarian sample and viable follicles were analyzed as a proportion of the total number of follicles counted. To avoid duplication of follicle numbers, only the follicles with nuclei present in the section were counted. If a follicle did not have a nucleus present on any of the sections, the relative position of the follicle was noted to ensure the follicle was counted only once. Follicular classifications were determined based on previously described classifications [1], [27]. Representative follicle types in this study are depicted in Figure 1.

### Statistical Analyses

All vaginal smear data and follicular analyses were recorded and analyzed blind with respect to treatment. Prior to vaginal cytology analyses and follicular analyses, all animals were randomly reassigned a new identification number, which was not disclosed to the person observing the slides or performing estrous stage classification.

Estrous cycle length was determined by counting the number of days from proestrus to the next proestrus [15], [28], [29]. The 75 days of post ovarian transplantation vaginal smears were divided into three blocks of 25 days: Block 1 (days 1–25 after surgeries), Block 2 (days 26–50 after surgeries), and Block 3 (days 51–75 after surgeries). The mean estrous cycle length across treatment groups was analyzed for all 75 days and for each block. In addition, the onset of estrous cycle was determined by the number of days after surgery to the first proestrus. Finally, the estrous cycles from each animal were classified into three categories: regular (4–5 days in length with either 1–2 days of estrus or 2–3 days of diestrus), extended (3–4 consecutive days of estrus or 4–5 consecutive days of diestrus), and abnormal (more than 4 consecutive days of estrus or more than 6 consecutive days of diestrus) [28].

Estrous cycle lengths (days) for all 75-days post-surgery and the onset of estrous cycle (days) across treatment groups were compared using Kruskal-Wallis ANOVA as the data failed the Shapiro-Wilk Test for normality. The estrous cycle lengths (days) for the three blocks were rank transformed and analyzed using MANOVA as the data were not normally distributed as determined by the Shapiro-Wilk Test. Whether or not individuals from each treatment group cycled until the end of the study was compared using a Chi-square Test of Independence. The differences in the number of regular, extended, and abnormal cycles between treatments were rank transformed and compared using MANOVA as the data failed the Shapiro-Wilk Test for normality. Classifications of estrous cycles were also compared using the Chi-square Test of Independence for differences in the percentage of females between treatments that exhibited abnormal cycles *versus* those that did not. A separate Chi-square Test of Independence was used to test the differences in the percentage of females between treatments that did and did not exhibit extended cycles. Percentage of viable follicles and the total number of follicles counted per ovarian sample were compared using MANOVA. The cross sectional area per ovarian sample of each treatment group was compared using Kruskal-Wallis ANOVA as the data failed the Levene’s Test for Homogeneity of Variances.

## Results

### Estrous Cyclicity

**Figure 2:**
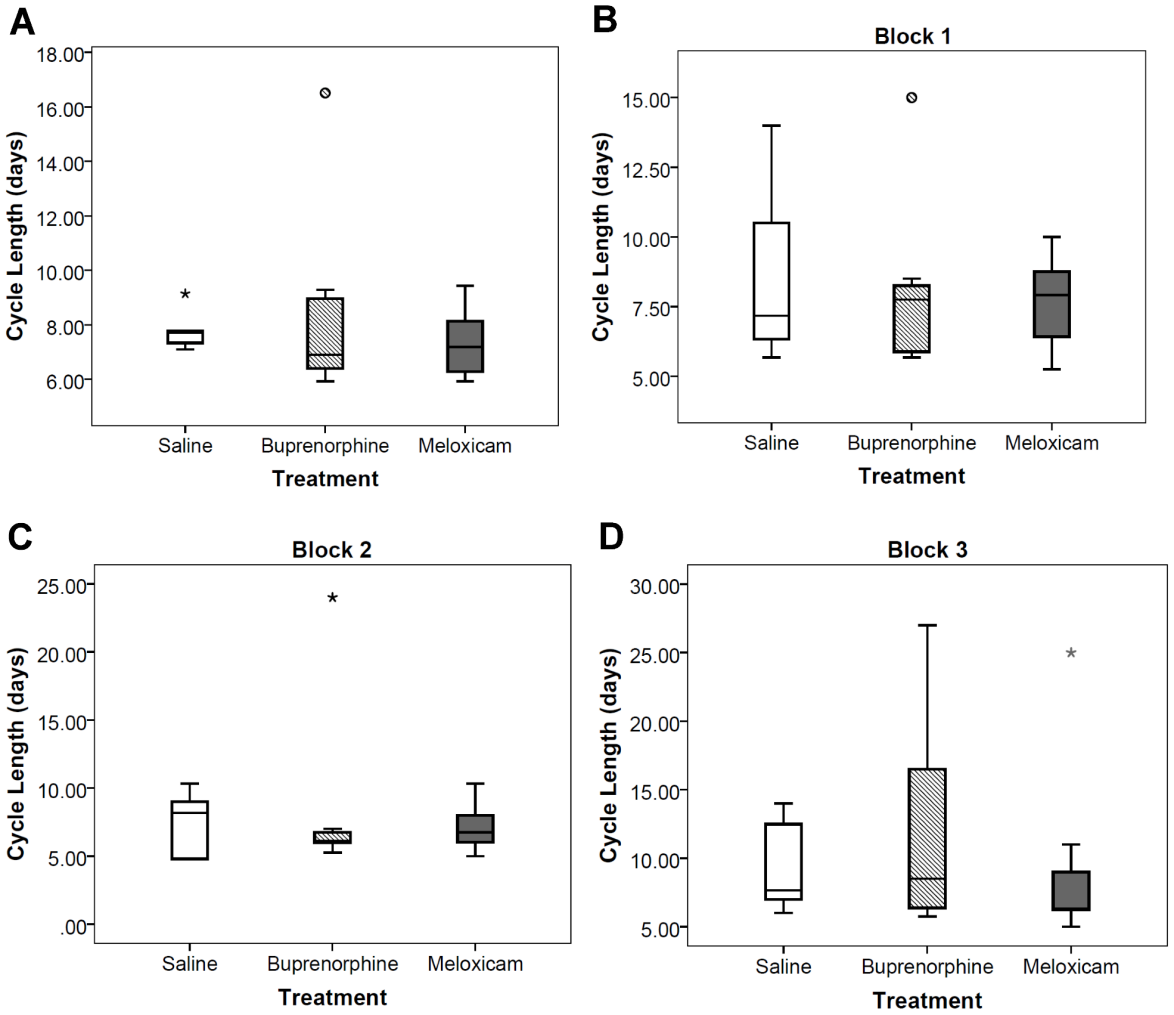
Boxplots of the estrous cycle length in days taken from daily vaginal cytology for each time block after surgery. (A) all 75 days (Kruskal-Wallis ANOVA: χ^2^ = 0.92, df = 2, *p* = 0.63). (B) block 1, days 1–25 after surgery. (C) block 2 days 26–50 after surgery. (D) block 3, days 51–75 after surgery. *N* = 22, saline (*n* = 6), buprenorphine (*n* = 8), meloxicam (*n* = 8). (Rank transformed, MANOVA: F(6,34) = 0.27, *p* = 0.95). The horizontal line in each box interior represents the median, the upper and lower whiskers show the maximum and minimum values, the upper hinge represents the 75^th^ percentile, the lower hinge represents the 25^th^ percentile, “o” represents an outside value, and asterisk (*) represents a far outside value.

Estrous cycle length was not significantly different across groups (Kruskal-Wallis ANOVA: χ^2^ = 0.92, df = 2, *p* = 0.63; Figure 2A). The median estrous cycle length for the saline-control was 7.7 days (*n* = 6, Q_1_ = 7.3, Q_3_ = 8.1), for the buprenorphine-treated was 6.9 days (*n* = 8, Q_1_ = 6.2, Q_3_ = 9.1), and for the meloxicam-treated was 7.2 days (*n* = 8, Q_1_ = 6.1, Q_3_ = 8.3). One female from each treatment group was excluded from analysis due to abnormal cycle lengths (>3 standard deviations from the sample mean).

The cycle lengths during Block 1 (days 1–25 after surgeries), Block 2 (days 26–50), and Block 3 (days 51–75) were not significantly different across treatment groups in any of the three time blocks (Rank transformed, MANOVA: F(6,34) = 0.27, *p* = 0.95; Figure 2B, 2C, 2D). The median cycle length during Block 1 was 7.2 days for the saline-control *(n* = 6, Q_1_ = 6.2, Q_3_ = 11.4), 7.8 days for the buprenorphine-treated (*n* = 8, Q_1_ = 5.8, Q_3_ = 8.4), and 7.9 days for the meloxicam-treated (*n* = 8, Q_1_ = 6.4. Q_3_ = 8.9). During Block 2, the median cycle length was 8.2 days for saline (Q_1_ = 4.8, Q_3_ = 9.3), 6.1 days for buprenorphine (Q_1_ = 6.0, Q_3_ = 6.9), and 6.8 days for meloxicam (Q_1_ = 5.9, Q_3_ = 8.0). During Block 3, the median cycle length was 7.7 days for saline (Q_1_ = 6.8, Q_3_ = 12.9), 8.5 days for buprenorphine (Q_1_ = 6.2, Q_3_ = 18.8), and 6.3 days for meloxicam (Q_1_ = 6.3, Q_3_ = 10.0). One female from each treatment group was excluded from analysis due to no cycling in at least one of the blocks.

There was no significant difference in the time until the onset of estrous cycling between groups (Kruskal-Wallis ANOVA: χ^2^ = 3.64, df = 2, *p* = 0.16). Median number of days to the onset of cycling was 9.0 days for the saline-control (*n* = 7, Q_1_ = 7.00, Q_3_ = 12.00), 6.0 days for the buprenorphine-treated (*n* = 9, Q_1_ = 5.00, Q_3_ = 9.00), and 7.0 days for the meloxicam-treated (*n* = 9, Q_1_ = 5.50, Q_3_ = 7.50). The onset of cycling was seen as early as 5 days post-surgery and the mean onset of estrous cycling across all treatment groups was 10 days (*SD* = 10.52) post-surgery. All females across all treatment groups exhibited estrous cyclicity after ovarian transplantation indicating successful transplantation for all recipient females.

Estrous cycle analysis showed no significant relationship between treatment and duration of cycling (whether or not a female cycled until the end of the study, Day 75) (Chi-square Test of Independence: χ^2^ = 3.87, df = 2, *p* = 0.15). Two females from the buprenorphine-treated group exhibited early cessation of cycling (approximately 20 days before sacrifice), while the other seven buprenorphine-treated females (77.8%) cycled until the end of the study. All females (100%) from the saline-control and meloxicam-treated groups cycled until the end of the study.

**Figure 3:**
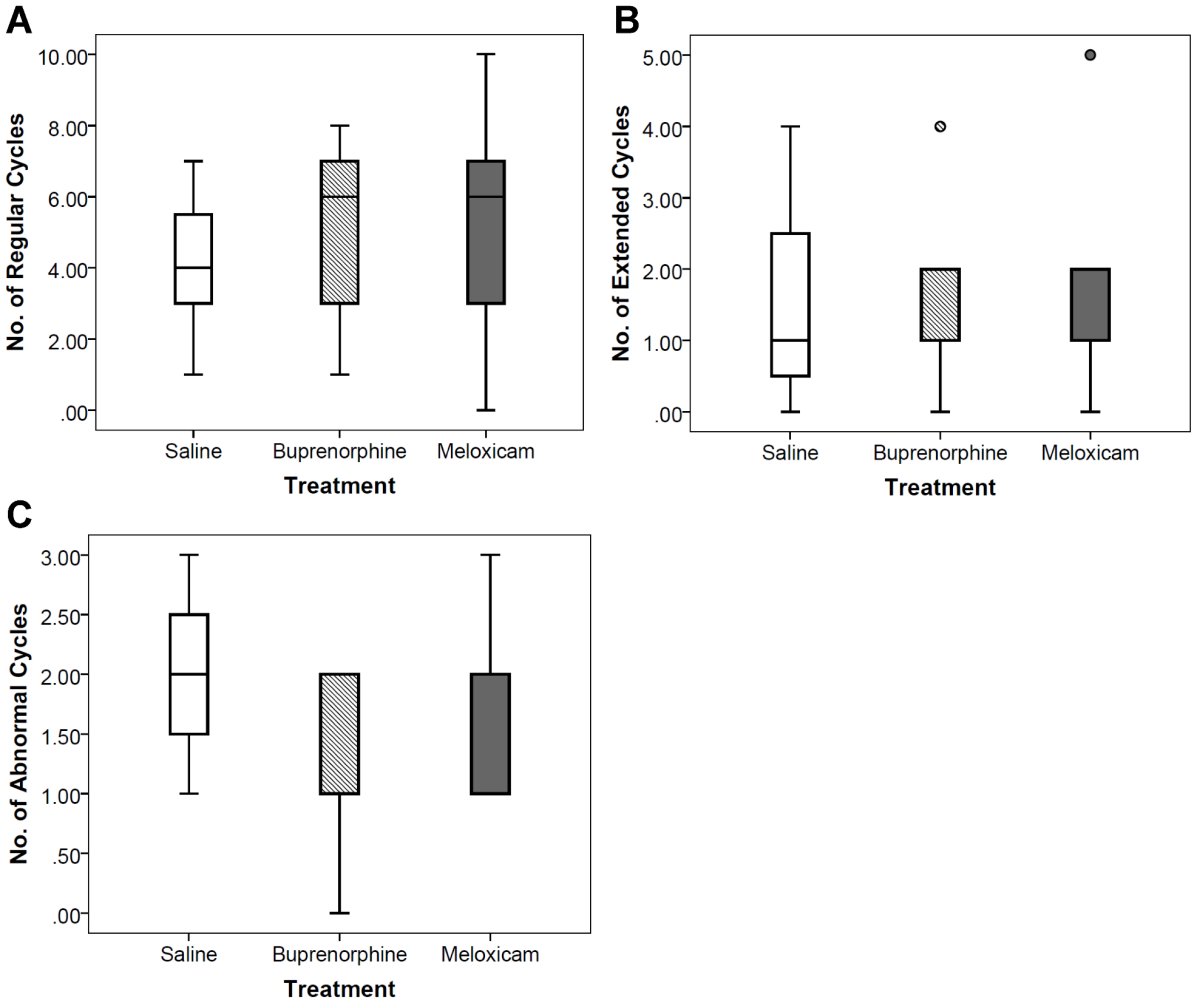
Boxplots of the estrous cycle classifications for each treatment. (A) regular, defined by 4–5 days in length. (B) extended, defined by 3–4 consecutive days of estrus or 4–5 consecutive days of diestrus. (C) abnormal, defined by having >4 days consecutive days of estrus or >6 consecutive days of diestrus. *N* = 25, saline (*n* = 7), buprenorphine (*n* = 9), meloxicam (*n* = 9). (Rank transformed, MANOVA: F(6,40) = 0.60, *p* = 0.73). The horizontal line in each box interior represents the median, the upper and lower whiskers show the maximum and minimum values, the upper hinge represents the 75^th^ percentile, the lower hinge represents the 25^th^ percentile, “o” represents an outside value, and asterisk (*) represents a far outside value.

There was no significant difference across treatments in the number of regular, extended, or abnormal cycles exhibited in the 75 days post-surgery (Rank transformed, MANOVA: F(6,40) = 0.60, *p* = 0.73; Figure 3). The saline group (*n* = 7) exhibited a median of 4 regular cycles (Q_1_ = 3.0, Q_3_ = 6.0), 1 extended cycle (Q_1_ = 0.0, Q_3_ = 3.0), and 2 abnormal cycles (Q_1_ = 1.0, Q_3_ = 3.0). The buprenorphine group (*n* = 9) exhibited a median of 6 regular cycles (Q_1_ = 3.0, Q_3_ = 7.0), 1 extended cycle (Q_1_ = 1.0, Q_3_ = 2.0), and 2 abnormal cycles (Q_1_ = 0.5, Q_3_ = 2.0). The meloxicam group (*n* = 9) exhibited a median of 6 regular cycles (Q_1_ = 3.0, Q*3* = 6.0), 1 extended cycle (Q_1_ = 0.5, Q_3_ = 2.0), and 2 abnormal cycles (Q_1_ = 1.0, Q_3_ = 2.5).

All females in the saline-control (*n* = 7) group and the meloxicam-treated group (*n* = 9) exhibited abnormal estrous cycles (more than 4 consecutive days of estrus or more than 6 consecutive days of diestrus) compared with seven females (77.8%) in the buprenorphine-treated (*n* = 9) group. The percentages of each treatment exhibiting abnormal estrous cycles as opposed to not exhibiting abnormal cycles were not significantly different (Chi-square Test of Independence: χ^2^ = 3.87, df = 2, *p* = 0.15, Table 1). Five saline-control, seven buprenorphine-treated, and seven meloxicam-treated females exhibited extended estrous cycles (3–4 consecutive days of estrus or 4–5 consecutive days of diestrus). The proportions of each treatment group exhibiting extended estrous cycles was also not significant (Chi-square Test of Independence: χ^2^ = 0.11, df = 2, *p* = 0.95, Table 1). All saline-control and buprenorphine-treated females and eight meloxicam-treated females exhibited at least one regular estrous cycle (Chi-square Test of Independence: χ^2^ = 1.85, df = 2, *p* = 0.40, Table 1).

**Table 1:** Percentage of females from each treatment exhibiting the three categories of estrous cycles: regular, extended, and abnormal.

Treatment	n	Females with Regular Cycles (%)	Females with Extended Cycles (%)	Females with Abnormal Cycles (%)
Saline	7	100	71.4	100
Buprenorphine	9	100	77.8	77.8
Meloxicam	9	88.9	77.8	100

Regular cycles defined by cycle lengths of 4–5 days, extended cycles defined by 3–4 days of consecutive estrus or 4–5 days of diestrus, and abnormal cycles defined by >4 days or consecutive estrus or >5 days of diestrus, (Chi-square Test of Independence, Regular χ^2^ = 1.85 and *p* = 0.40, Extended χ^2^ = 0.11 and *p* = 0.95, Abnormal χ^2^ = 3.87 and *p* = 0.15).

### Histological Analysis

**Figure 4:**
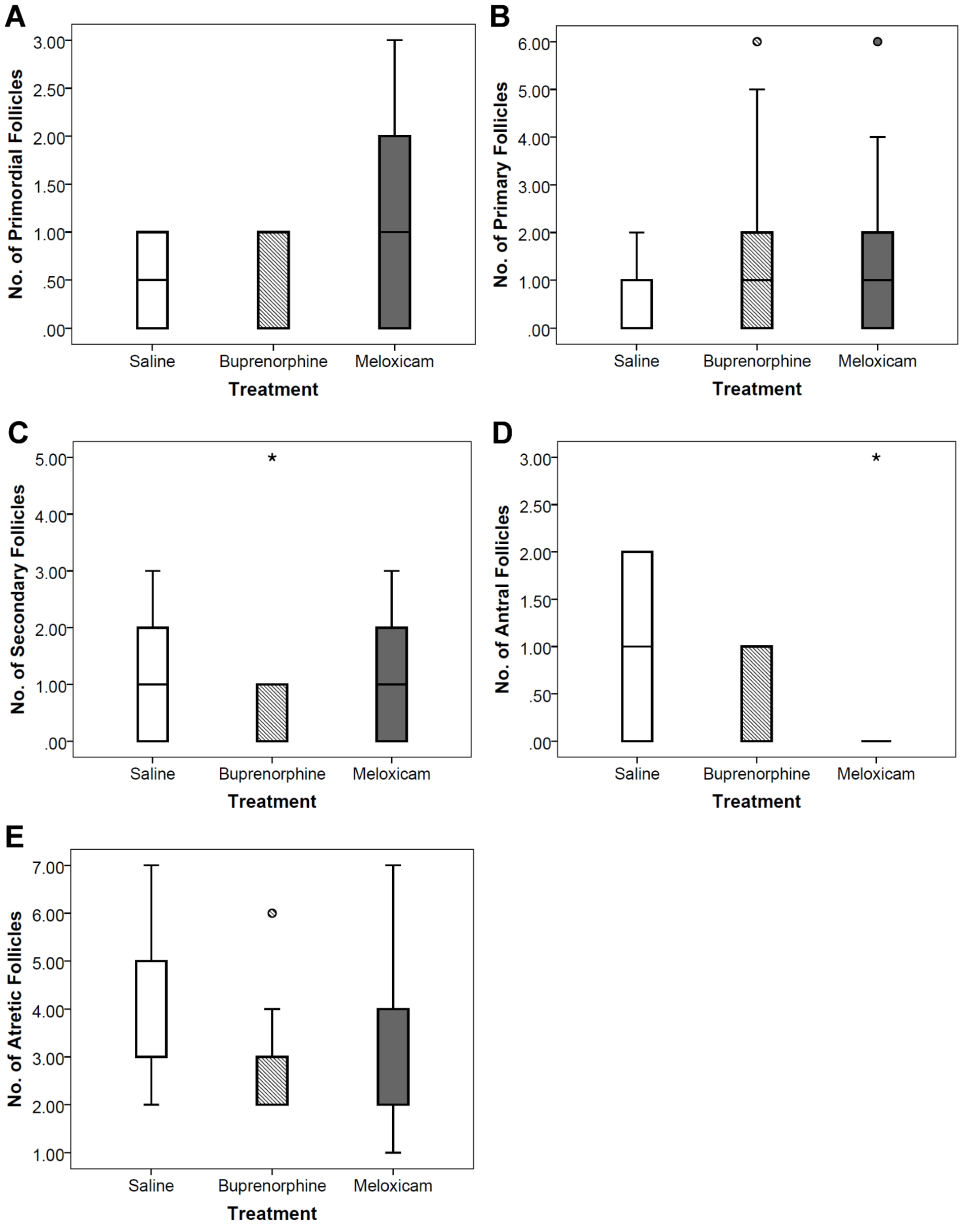
Boxplots of the number of each follicle type per ovarian sample (each ovarian sample consists of nine tissue sections). *N* = 24, saline (*n* = 6), buprenorphine (*n* = 9), meloxicam (*n* = 9). (Rank transformed, MANOVA: F(10,34) = 0.45, *p* = 0.91). (A) primordial follicles. (B) primary follicles. (C) secondary follicles. (D) early antral follicles. (E) antral follicles. (F) atretic follicles. The horizontal line in each box interior represents the median, the upper and lower whiskers show the maximum and minimum values, the upper hinge represents the 75^th^ percentile, the lower hinge represents the 25^th^ percentile, “o” represents an outside value, and asterisk (*) represents a far outside value.

One female from the saline-control group was excluded from all histological analyses, as tissue sections from this female were lacking ovarian tissue. The number of each follicle type across treatment groups was not significantly different (Rank transformed, MANOVA: F(10,34) = 0.45, *p* = 0.91; Figure 4). Treatment did not have any significant effect on the proportion of viable follicles and the number of total follicles observed per ovarian sample (MANOVA: F(4,40) = 0.10, *p* = 0.98; Table 2). The mean proportion of viable follicles was approximately 47.8% for the saline-control group, 43.7% for the buprenorphine-treated group, and 50.9% for the meloxicam-treated group. The mean total number of follicles (including viable and atretic follicles) counted from each ovarian sample was 7.3 for the saline-control, 6.9 for the buprenorphine-treated, and 7.6 for the meloxicam-treated groups.

**Table 2:** The proportion of viable follicles and the total number of follicles per ovarian sample for each treatment.

Treatment	n	Proportion of Viable Follicles perovarian sample	Total No. of Follicles per ovarian sample
Saline	6	0.48±0.18	7.3±2.07
Buprenorphine	9	0.44±0.26	6.9±5.09
Meloxicam	9	0.51±0.24	7.6±4.33

Data are shown as mean ± SD. Each ovarian sample consists of nine tissue sections. The total number of follicles per ovarian sample includes counts from viable and atretic follicles. (MANOVA, F(4,40) = 0.10, *p* = 0.98).

No significant differences were observed in the cross-sectional area of the ovary across treatments (Kruskal-Wallis ANOVA: χ^2^ = 0.76, df = 2, *p* = 0.69). The median cross sectional area of the saline-control group was 1.33 mm^2^ (*n* = 6, Q_1_ = 0.91, Q_3_ = 1.62), of the buprenorphine-treated group was 1.64 mm^2^ (*n* = 9, Q_1_ = 0.91, Q_3_ = 2.00), and of the meloxicam-treated group was 1.40 mm^2^ (*n* = 9, Q_1_ = 1.30, Q_3_ = 1.69).

## Discussion

Indicators of ovarian transplant success, estrous cyclicity, and viability of follicles revealed no significant differences between treatment groups. Previous studies have found that both opiates and NSAIDs negatively affect angiogenesis with some conflicting results regarding increased or decreased angiogenesis with opiate treatments [6], [8], [9], [30], [31]. Most of these studies have employed high doses of analgesics for prolonged periods, which do not represent the veterinary recommended doses utilized in the current study [6], [8], [9], [27], [28]. The results from this study indicate that recommended doses for a moderate time period do not negatively affect the transplanted ovary in aged females beyond what is expected by the surgical process itself.

Neovascularization of an ovarian heterotopic autotransplant could be observed as soon as three days after transplantation in mice and two days in rats [32], [33], [34]. Restoration of ovarian cycling has been reported to occur as soon as 10–14 days after transplantation of whole ovary transplants and ovarian grafts [16], [17], [35]. However, after transplantation of whole ovaries in this study, as opposed to transplantation of ovarian grafts in previous studies, the onset of estrous cycle occurred as early as five days post-surgery with a mean of 10 days for all females.

Animal models of ovarian transplantations are especially sensitive to the effects of aging. In the present study, ovaries from 2-month old females were transplanted into 11-month old females. The CBA/J mice used in the current study have a lifespan of 647 days [36] with a decline in fecundity at approximately 11 months of age [37]. Total follicle numbers in young 6–8 week old C3H/HeNCrlBR and B6129SF1/J ovaries that had been grafted into 6–8 week old B6C3F1/J and B6129SF1/J recipients was lower than intact ovary total follicle numbers and have been estimated to be about 100 follicles per ovary [16] due to the hypoxia experienced with ovarian transplantation [5]. Also of importance is that gonadotropin levels decrease with age, reducing gonadotropic input to the ovaries [38], [39]. In middle-aged rodents, a significant decrease was seen in the levels of LH during an LH surge as well as a delayed occurrence of the LH surge compared with young rodents [39]. GnRH levels may not change with age, but the LH response to GnRH decreases with age [38]. These age-associated factors could lead to decreased follicular development and thus to decreased follicular counts in the aged recipients of this study. These factors may have contributed to the early cessation of cycling seen in two of the buprenorphine-treated animals. It is also possible that those two animals experienced decreased vascularization of the transplanted ovary but it is unclear whether this can be attributed to the analgesic buprenorphine.

The effects of analgesics on ovarian transplant success in aged females require further investigation. This study suggests that at the recommended veterinary doses [12], [24], [25], [26], the two analgesics studied do not decrease angiogenesis to the extent that there are alterations in estrous cycle length or the proportion of viable follicles and the total number of follicles per ovary. This would imply that the veterinary recommended doses of analgesic do not induce additional damage after ovarian transplantation nor significantly affect the function of the transplanted ovary. Thus, the recommended dosages for buprenorphine and meloxicam may be beneficial to decrease surgical pain in mice without negatively affecting ovarian transplant survival as assessed by estrous cycle length and follicle count.
